# Sexual and temporal variations in floral scent in the subdioecious shrub *Eurya japonica* Thunb

**DOI:** 10.1002/ece3.4378

**Published:** 2018-07-22

**Authors:** Hui Wang, Peiming Zheng, Dan Aoki, Takashi Miyake, Sachie Yagami, Yasuyuki Matsushita, Kazuhiko Fukushima, Michiko Nakagawa

**Affiliations:** ^1^ School of Life Science Shandong University Jinan China; ^2^ Graduate School of Bioagricultural Sciences Nagoya University Nagoya Japan; ^3^ Structural Materials Research Institute National Institute of Advanced Industrial Science and Technology Nagoya Japan; ^4^ Faculty of Education Gifu University Gifu Japan

**Keywords:** dynamic headspace method, floral scent, GC–MS, sexual dimorphism, subdioecious shrub

## Abstract

In many flowering plants, floral scents are a significant trait for visitors, playing an important role in attracting pollinators and/or detracting herbivores. The evolution of flowering plants from hermaphroditism to dioecy is often accompanied by sexual dimorphism in floral scent. In this study, floral scents emitted by different sexual morphs of the subdioecious shrub *Eurya japonica* Thunb. were collected using a dynamic headspace method, and sexual and temporal variations were evaluated by gas chromatography–mass spectrometry (GC–MS). Two volatiles, α‐pinene and linalool, were identified as the major components of floral scents in females, hermaphrodites, and males. The males emit higher amounts of floral scents, particularly α‐pinene, compared to females or hermaphrodites. Floral scents emitted by males generally decrease as flowers enter senescence, whereas those from females or hermaphrodites do not significantly differ. Intraspecific variations in floral scents of subdioecious species provided by this study would contribute to better understanding of sexual dimorphism in floral scent.

## 1 INTRODUCTION

As one of significant traits for visitors, floral scents in many flowering plants play an important role attracting pollinators and/or detracting herbivores (Custódio, Serra, Nogueira, Gonçalves, & Romano, 2006; Dötterl, Glück, Jürgens, Woodring, & Aas, 2014; Miyazawa et al., 2016; Tsuji & Sota, 2013). Floral scent signatures are composites of volatile chemicals at specific stoichiometric concentrations (Knudsen, Tollsten, & Bergstrom, 1993; Wright & Schiestl, 2009) that are produced by floral tissues (Dudareva & Pichersky, 2006). The types and concentrations of floral scents affect the interaction between plants and visitors (Ayasse et al., 2000; Wright, Thomson, & Smith, 2005). Several thousands of compounds from various floral scents have been identified (Knudsen et al., 1993; Ohloff, 1994). Typically, these compounds are “fatty acid derivatives, benzenoids, phenylpropanoids, isoprenoids, and nitrogen‐ and/or sulfur‐containing compounds” (Knudsen et al., 1993), with characteristics of “low polarity, and slight water solubility, as well as high vapor pressure and lipophilicity” (Schade, Legge, & Thompson, 2001).

The evolution of flowering plants from combined sexes (hermaphroditism) to separate sexes (dioecy) is often accompanied by sexual dimorphism in floral scent (Ashman, 2009). Previous studies have shown that floral scents can differ qualitatively and quantitatively among various sexual flowers (Dötterl & Jürgens, 2005; Miyazawa et al., 2016) and flower parts (Dötterl & Jürgens, 2005) or same flowers at different flowering stages (Custódio et al., 2006; Kumano & Ymaoka, 2006; Schade et al., 2001). Moreover, environmental conditions (e.g., temperature, humidity, light, and time of the day) may determine the quality and quantity of floral scents (Azuma, Toyota, & Asakawa, 2001; Custódio et al., 2006; Kumano & Ymaoka, 2006).

The majority of previous investigations have focused on dioecious species and compared the differences in floral scents between females and males (Dötterl et al., 2014; Dufa, Hossaert‐McKey, & Anstett, 2004; Milet‐Pinheiro et al., 2015; Tollsten & Knudsen, 1992; Tsuji & Sota, 2010). Ashman, Bradburn, Cole, Blaney, and Raguso (2005) investigated emission rates and floral scent composition in a gynodioecious plant (*Fragaria virginiana*) in females and hermaphrodites. In this study, floral scent differences in a subdioecious sexual system (females, hermaphrodites, and males) were investigated. Resource reallocation favors the evolution of sexual dimorphism (Charlesworth, 1999). The sexual selection theory (Bateman, 1948; Dötterl et al., 2014) predicts that “males are limited in their reproductive success by access to mates, whereas females are more limited by resources” (Waelti, Page, Widmer, & Schiestl, 2009). Thus, males in the majority of species emit more scents per flower than females to attract visitors (reviewed in Ashman, 2009). Therefore, it is of much interest to investigate whether the profile and the temporal pattern of floral scents of hermaphrodites are similar to that in males or females in subdioecious species. This will improve our understanding of the patterns of sexual dimorphism in floral scent.

To better understand the patterns of sexual dimorphism in floral scent, this study aimed to i) chemically characterize its floral scents, and to assess ii) qualitative and iii) temporal variations in floral scents in female, hermaphrodite, and male flowers.

## 2 MATERIALS AND METHODS

### 2.1 Plant materials

*Eurya japonica* Thunb., an evergreen broadleaf understory tree belonging to the Pentaphylaceae family (approximately 0.5–3 m in height) (Tsuji & Sota, 2013; Wang, Matsushita, Tomaru, & Nakagawa, 2016), was investigated in this study. As a subdioecious species, *E. japonica* consists of three sexual morphs: males with staminate flowers, females with pistillate flowers, and hermaphrodites with perfect flowers (i.e., flowers with both stamens and pistils) as well as staminate flowers and/or pistillate flowers (Motooka et al., 2015; Tsuji & Sota, 2013; Wang, Matsushita, Tomaru, & Nakagawa, 2015, 2017; Wang et al., 2016). In Japan, flowers bloom from late February to early April, lasting for about 2 weeks (Kitamoto, Takasu, & Yagi, 1992; Wang et al., 2015). Generally, the flowers of an individual open simultaneously. It bears a few to several hundred small flowers depending on the age, height, and microhabitat conditions (Miyazawa et al., 2016). Flower diameter and length are approximately 2–5 mm and 3–5 mm, respectively. *E. japonica* is an insect‐pollinated plant, its pollinators are mainly dipteran and thysanopteran insects (Kitamoto et al., 1992; Tsuji & Ohgushi, 2018), and its florivores are generally lepidopteran and dipteran larvae and hemipteran insects (Tsuji & Sota, 2013).

The plants of *E. japonica* used in this study are situated in the Higashiyama Campus of Nagoya University, Nagoya, Japan (35°10′N, 136°58′E, 55–80 m a.s.l.). The experiments were conducted from early to the end of March 2016.

To investigate sexual and temporal variations in floral scents during flowering, seven female individuals, six hermaphrodites, and seven males were selected as targets. Before flowering, three flower branches (with leaves and flowers, approximately 30 cm in length) and three leaf branches (with only leaves, approximately 30 cm in length) in each individual were selected and bagged in nylon mesh bags to avoid visitation. The branches were successively harvested at three stages: stage 1 (initial stage, all flowers of the entire tree had bloomed), stage 2 (developing stage, 6 or 7 days after the initial stage), stage 3 (senescence stage, 11 or 12 days after the initial stage); and 1 flower branch and 1 leaf branch were harvested from each individual at each stage. Harvesting was conducted at 9:00–11:00 a.m. The number of flowers in each flower branch was recorded: females, 72 ± 34 (mean ± *SD*,* n* = 21, i.e., 3 stages × 7 individuals); hermaphrodites, 67 ± 30 (*n* = 18, 3 × 6); males, 68 ± 34 (*n* = 21, 3 × 7).

### 2.2 Flower scents collection

**Figure 1:**
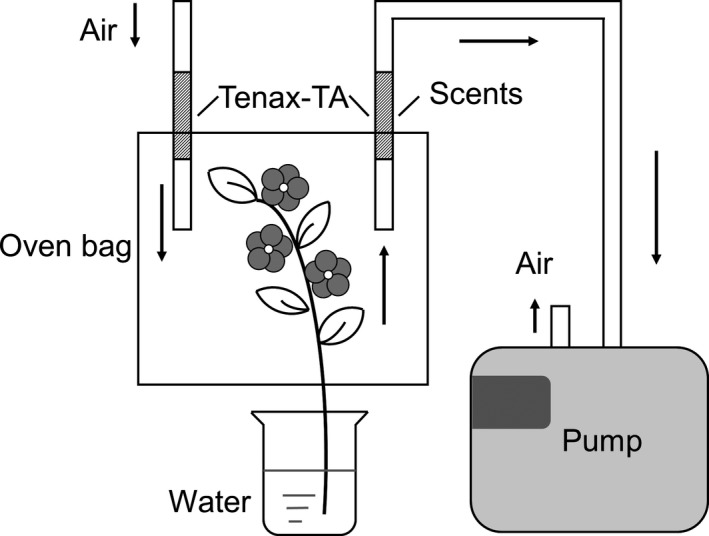
Schematic diagram of floral scent collection device using dynamic headspace method

Scents were collected in the laboratory with a pump extractor using a dynamic headspace method (Dötterl & Jürgens, 2005; Figure 1). To maintain freshness, the flower or leaf branches were harvested and immersed in water at room temperature (shown in Figure 1), immediately followed by scent collection. The branches were enclosed in a polyethylene oven bag (340 mm × 240 mm), and the emitted volatiles were trapped in an adsorbent tube using a membrane pump (SIBATA, Inc., Akashi, Japan). The flow rate was adjusted to 200 ml/min using a flow meter. Samples were collected for 1 hr. An adsorbent tube was constructed using a PTFE tube (Φ3 × 5 mm, 100 mm) that was filled with 60 mg of Tenax‐TA (60–80 mesh). The adsorbents were fixed in the tubes using glass wool. Room air was simultaneously collected and used as control.

The volatiles trapped in the adsorbent tube were dissolved and washed with diethyl ether (5 ml × 3) and collected into a test tube. Docosane (0.1 g/L, 0.1 ml) was used as internal standard. The collected liquid was concentrated to approximately 1.5 ml by N_2_ and stored at 4°C.

### 2.3 Gas chromatography–mass spectrometry (GC–MS)

The collected volatiles were measured using GC–MS (QP 2010, Shimadzu, Kyoto, Japan), which was equipped with a capillary column (Rxi–1 m, 30 m × 0.32 mm i.d. and a film thickness of 0.25 μm). GC–MS‐operating conditions were as follows: injector temperature, 250°C; oven temperature program, 35°C held for 5 min, 35°C→180°C (5°C/min), then 180°C→200°C (10°C/min), and then held for 10 min, and finally to 280°C (20°C/min) and then held for 5 min; carrier gas, He; flow rate, 1.6 ml/min; interface temperature, 250°C; and ion source temperature, 200°C. The quantity of each volatile compound was calculated by comparing the GC data with the internal standards.

### 2.4 Statistical analysis

The *Kruskal*–*Wallis* test was used to assess differences in the amount of each volatile between the flower branch and leaf branch of females, hermaphrodites, and males. The generalized linear mixed models (GLMMs) were used to examine the effects of sexual morphs (female, hermaphrodite, and male) and flowering stages (stage 1, 2, and 3) on the amount of floral scents (Bolker et al., 2009). In the models, the sexual morphs, flowering stages, and their interactions were set as fixed effects, and target individuals were set as random effects. To assess the statistical significance of each fixed factor, the changes in deviance when each factor was removed from the full model were compared with the *F*‐test for Gaussian error distributions with Identity link functions (Bolker et al., 2009). The data on the amount of each volatile were log‐transformed before statistical analysis. Post hoc comparisons were conducted by adjusting family‐wise errors based on Tukey's method at *p *=* *0.05. Statistical analyses were performed using the R 3.4.3 software (R Development Core Team, 2017; *nlme, lme4, Mass, and multcomp packages*).

## 3 RESULTS

### 3.1 Identification of major floral scents

**Figure 2:**
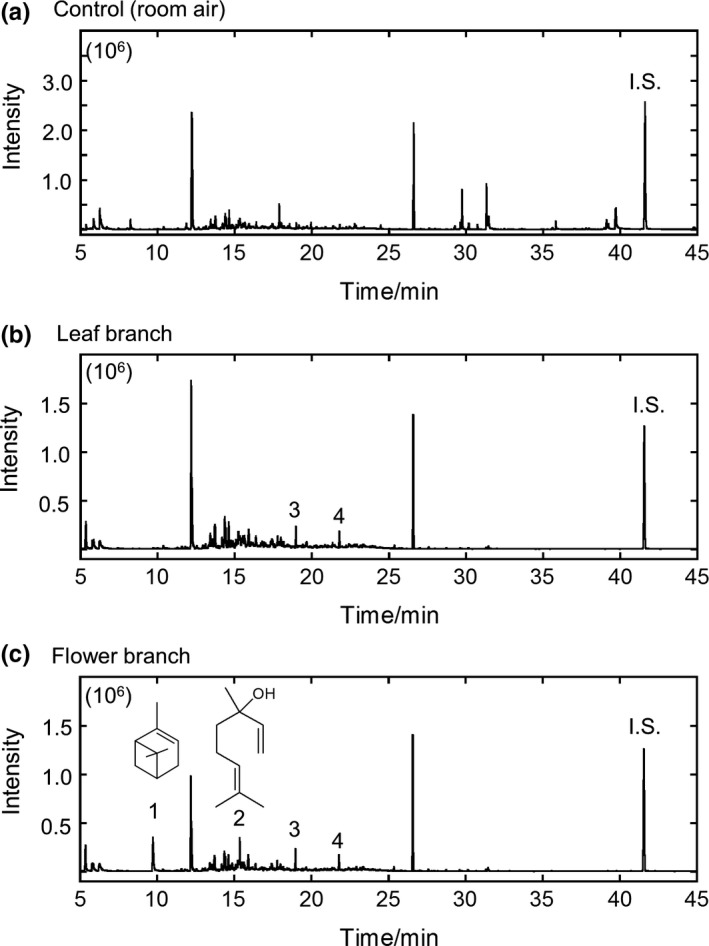
Total ion chromatogram (TIC) of the collected volatiles from room air (a), leaf branch (b), and flower branch (c). The chemical structures of peaks 1 (α‐pinene) and 2 (linalool) are shown. I.S., internal standard (docosane)

Figure 2 shows the total ion chromatograms (TICs) of the collected volatiles and room air (control). The peak assignments of the main compounds were based on the mass spectral data of previous studies (Adams, 2007; Motooka et al., 2015) and standard chemicals under consideration of the retention time (RT). The structures of peaks 1 and 2 are illustrated in Figure 2c.

**Figure 3:**
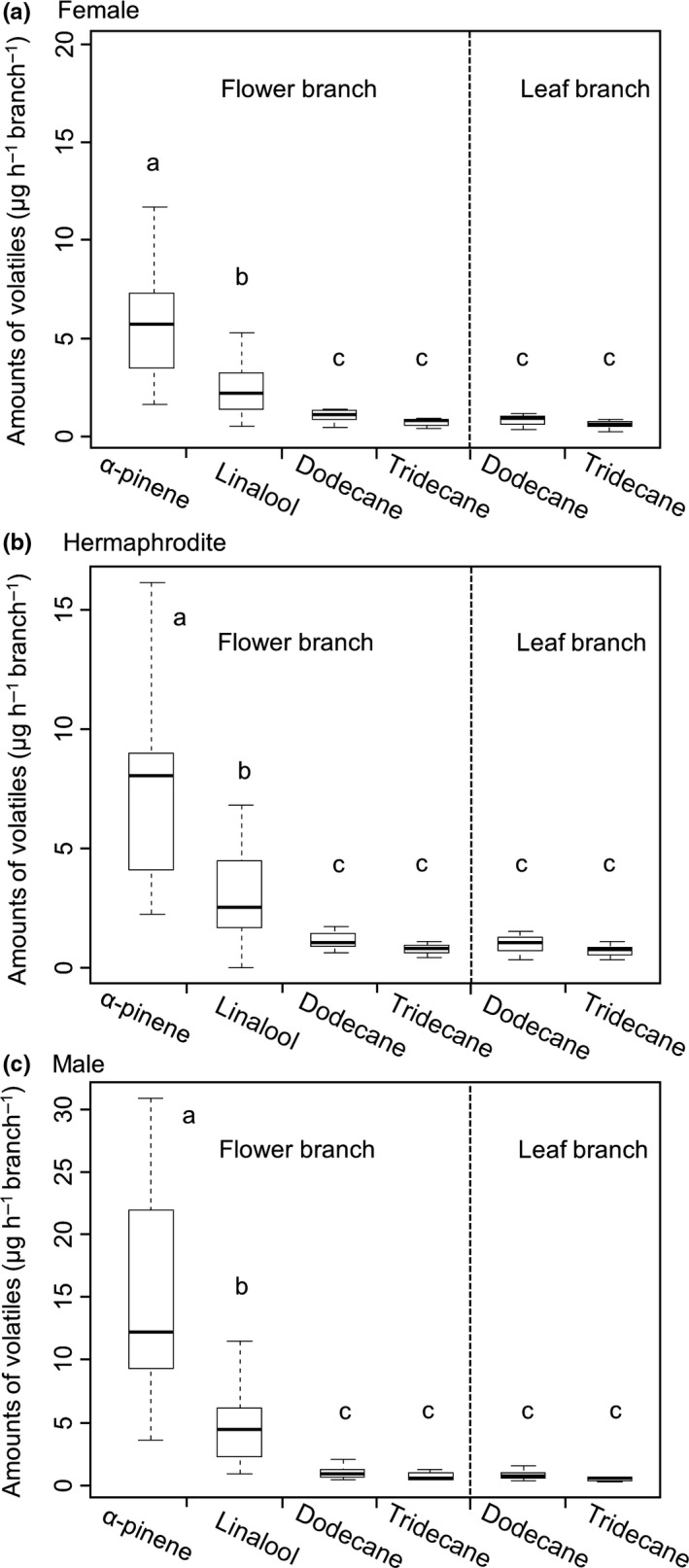
Amounts (μg·hr^−1^·branch^−1^) of major volatiles emitted by flower and leaf branches of different sexual morphs at the entire flowering stages. Maximum and minimum values for each sample are shown at the upper and lower ends of the vertical bars, respectively. The 75% and 25% points are given by the upper and lower ends of the box, respectively. The middle bar indicates the median (female: *n* = 21, 3 stages × 7 individuals; hermaphrodite: *n* = 18, 3 × 6; male: *n* = 21, 3 × 7). Different letters beside the bars indicate significant differences in the results of multiple comparisons in which family‐wise errors were adjusted using Tukey's method at *p *=* *0.05

Four compounds were detected in the flower branches of the three sexual morphs or flowering stages: α‐pinene, linalool, dodecane, and tridecane (Figure 2c). α‐Pinene and linalool are terpenoids, whereas dodecane and tridecane are aliphatics. For the entire flowering period (stage 1, 2, and 3), the amount (μg·hr^−1^·branch^−1^) of α‐pinene or linalool emitted by flower branches is significantly higher (*p *<* *0.01) than that of dodecane or tridecane in all three sexual morphs (Figure 3). The predominant volatiles consist of terpenoids (α‐pinene and linalool) with percentages of 85.8% ± 12.0% (*n* = 21, 3 stages × 7 individuals), 86.9% ± 12.0% (*n* = 18, 3 × 6), and 94.3% ± 8.2% (*n* = 21, 3 × 7) in females, hermaphrodites, and males, respectively.

### 3.2 Sexual variations in floral scent

The amount of floral scents (α‐pinene and linalool) was significantly affected by the sexual morphs (female, hermaphrodite, and male), flowering stages (stage 1, 2, and 3), and their interaction (Table 1).

**Table 1:** Effect of sexual morphs (female, hermaphrodite, and male), flowering stages (stage 1, 2, and 3), and their interactions on the amount of α‐pinene and linalool. To test the statistical significance of explanatory variables, the changes in deviance when each variable was removed from the full model were compared with *F* distributions for Gaussian distributions. Boldface indicates statistical significance

Response variables	Explanatory variables	*df*	*F*	*p*
α‐Pinene	Sexual morphs	2	8.122	**<0.01**
	Flowering stages	2	6.984	**<0.01**
	Sexual morphs × flowering stages	4	2.803	**<0.05**
Linalool	Sexual morphs	2	1.834	0.190
	Flowering stages	2	12.000	**<0.001**
	Sexual morphs × flowering stages	4	1.344	0.279

**Figure 4:**
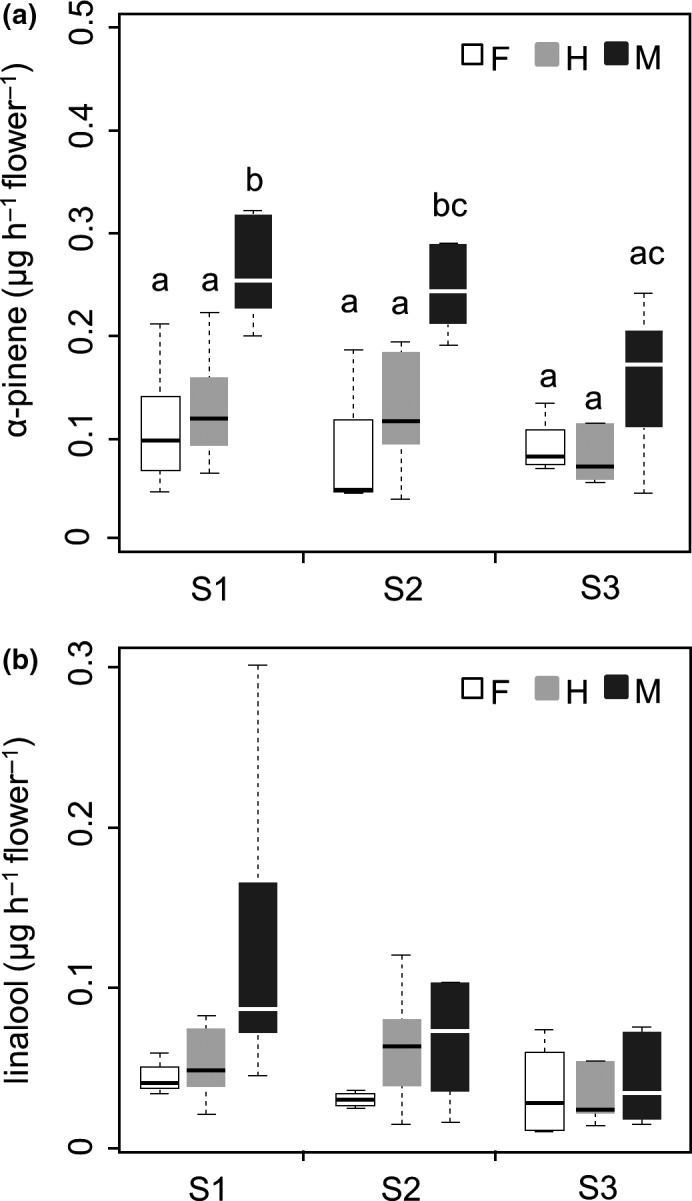
Amounts (μg hr^−1^ flower^−1^) of main volatiles of floral scents emitted by different sexual morphs or flowering stages (female: *n* = 7; hermaphrodite: *n* = 6; male: *n* = 7). Different letters beside the bars indicate significant differences in the results of multiple comparisons in which family‐wise errors were adjusted using Tukey's method at *p *=* *0.05. F, female; H, hermaphrodite; M, male; S1, stage 1; S2, stage 2; and S3, stage 3

At stage 1 (Figure 4a), males emit significantly higher amounts of α‐pinene (μg·hr^−1^·flower^−1^) compared to females or hermaphrodites in post hoc comparisons. In addition, males emitted marginally higher amounts of linalool (0.13 ± 0.09) than females (0.05 ± 0.02) or hermaphrodites (0.05 ± 0.02), although this difference was not statistically significant (Figure 4b). However, no significant difference in the amount of α‐pinene/linalool between females and hermaphrodites was observed. A similar situation was observed at stage 2 (Figure 4).

At stage 3 (Figure 4a), males emitted marginally higher amounts of α‐pinene (μg·hr^−1^·flower^−1^) (0.16 ± 0.07) than females (0.09 ± 0.02) or hermaphrodites (0.11 ± 0.08), whereas almost no difference between females and hermaphrodites was detected. No significant differences in the amount of linalool emitted by different sexual morphs were observed (Figure 4b).

### 3.3 Temporal variations in floral scent

Figure 4 reveals that the amount of floral scents (α‐pinene or linalool) emitted by females or hermaphrodites does not significantly differ during flowering (from initial to senescence stages). Conversely, the amount of α‐pinene emitted by males at stage 3 is significantly lower than that at stage 1 (Figure 4a). Similarly, the amount of linalool emitted by males shows a decreasing trend from stages 1 to 3 (0.13 ± 0.09 vs. 0.06 ± 0.05), although these variations are not statistically significant.

## 4 DISCUSSION

### 4.1 Identification of major floral scents

Floral scents are generally composed of dozens, even hundreds of volatile chemicals (Knudsen et al., 1993; Miyazawa et al., 2016; Motooka et al., 2015; Tollsten & Knudsen, 1992). Motooka et al. (2015) and Miyazawa et al. (2016) prepared extracts from the flowers, vegetative parts, or flower buds of *E. japonica* in organic solvents, which were then analyzed using GC–MS. More than 50 compounds were detected in the essential oils. In the present study, only four major volatiles were detected. Differences in the results may be attributable to the use of various methods in these studies.

Under consideration of the results of the control (room air) and leaf branches (Figures 2 and 3), α‐pinene and linalool are the major components of floral scents, whereas dodecane and tridecane are emitted from leaves and/or other vegetative parts in all of the three sexual morphs and flowering stages.

### 4.2 Sexual variations in floral scent

The evolution from hermaphroditism to dioecy is coupled to sexual dimorphism in floral scent (Ashman, 2009). In this study, the floral scents of different sexual morphs show only quantitative (quantity of volatile compounds) differences and no qualitative (blend composition) differences (Figures 2, 3, 4). These findings indicate that the observed differences in floral scents between sexual morphs cannot be explained by the emission of additional pollen‐ or stigma‐specific compounds in flowers (Ashman et al., 2005; Mayo, Bogner, & Boyce, 1997; Miyake & Yafuso, 2003; Vogel, 1990).

In this study, the production of characteristic compounds of floral scents (particularly α‐pinene) in males is generally higher than that in females or hermaphrodites (Figure 4). Previous researches have reported similar results in some dioecious species (Dötterl & Jürgens, 2005; Dötterl et al., 2014; Waelti et al., 2009). Sexual selection theory predicts differential resource investment among different sexual morphs to attract pollinators (Bateman, 1948; Dötterl et al., 2014; Waelti et al., 2009). Accordingly, males should thus be selected to invest more resources in floral scents than females or hermaphrodites to enhance pollination success in subdioecious species, *E. japonica*.

### 4.3 Temporal variations in floral scent

Previous studies have revealed temporal variations in floral scents (e.g., Balao, Herrera, Talavera, & Dötterl, 2011; Dötterl, Jahreiss, Jhumur, & Jürgens, 2012; Kumano & Ymaoka, 2006; Miyake, Yamaoka, & Yahara, 1998). Kumano and Ymaoka (2006) showed that floral scent emissions in *Homalomena propinqua* increase from 06:00 a.m. to 09:00 a.m. on the first and second days of opening. Schade et al. (2001) reported that the steady‐state levels of 10 volatiles emitted by carnation flowers independently change as flowers develop and enter senescence. These reports thus suggest that the synthesis of floral scents is developmentally regulated.

In this study, floral scents emitted by males generally decrease as flowers undergo senescence (Figure 4), which agrees with the findings of previous studies (Tollsten, 1993; Tollsten & Bergström, 1989). The rapid decline in scent emission could thus be a mechanism of resource reallocation (Ashman et al., 2005) or strategy to avoid/reduce attacks by detrimental herbivores after pollination (Muhlemann, Waelti, Widmer, & Schiestl, 2006; Wright & Schiestl, 2009).

## CONFLICT OF INTEREST

None declared.

## AUTHOR CONTRIBUTIONS

HW, PZ, TM, and MN conceptualized and designed the study; HW and PZ conducted floral scent collection; HW, PZ, DA, SY, YM, and KF performed chemical analysis; and PZ, HW, TM, and MN wrote the manuscript. All authors have reviewed and approved the final manuscript.

## DATA ACCESSIBILITY

Data are available from the Dryad Digital Repository.
